# Importance and Relevance of Phytochemicals Present in *Galenia africana*

**DOI:** 10.1155/2022/5793436

**Published:** 2022-01-25

**Authors:** Dario Heredia, Ivan Green, Jeremy Klaasen, Farzana Rahiman

**Affiliations:** ^1^Department of Medical Bioscience, University of the Western Cape, Cape Town 7535, South Africa; ^2^Department of Chemistry, University of the Western Cape, Bellville 7535, Cape Town, South Africa; ^3^Skin Research Lab, Department of Medical Biosciences, University of the Western Cape, Cape Town 7535, South Africa

## Abstract

Many people in developing countries rely primarily on medicinal plants as their main source of healthcare, particularly for the treatment of skin infections. Despite the widespread use of medicinal plants, there is a lack of literature describing the relevance and risks of exposure of the phytochemicals present. *Galenia africana* has been used traditionally in the form of pastes, decoctions, and lotions to treat wounds and other skin-related ailments. This is a report on the phytochemical composition of *G. africana* and a review on the pharmacological importance and relevance of these phytochemicals. The major groups of phytochemicals identified in *G. africana* extracts were aliphatics, aliphatic triterpenoids, fatty acids, flavonoids, and phenolic and tocopherol compounds. These have been found to exhibit medicinal properties, thus highlighting the need to assess the safety of *G. africana* for topical application. The information related to the safety of the various compounds could indicate the potential risks related to accidental intake of the extract upon topical product applications. This report concludes that the quantities of the phytochemicals present in *G. africana* should not cause undue risk to human health, which provides comfort to pursue future work on using and developing *G. africana* as a therapeutic agent.

## 1. Introduction

Approximately 70–95% of the population in developing countries rely on medicinal plants as their primary source of healthcare [1]. These medicinal plants are highly sought after due to their apparent ability to treat burns and promote wound healing [1]. The use of such plants as a treatment option for skin infections has become common practice in many rural areas [2]. It has been reported that approximately 27 million people in South Africa rely on the use of medicinal plants to treat skin infections, in particular those caused by pathogens such as *Staphylococci* species and *Candida albicans* [3].

The *Galenia africana* plant species also known as ‘kraalbos' or ‘geelbos', belonging to the family Aizoaceae, is endemic to Southern Africa, where it is most commonly found in the Namaqualand region of South Africa, but has recently become more widespread in the Western and Southern Karoo [4–6]. Kraalbos is an aromatic, woody perennial sub-shrub which grows to a height of 0.5–1.5 m high, having oppositely arranged green leaves of about 5 cm long and hairless, which tend to change from green to yellow with age. The terminal ends of the twigs are the sites at which numerous small yellow flowers, about 1.5 mm in diameter, are born in large loose heads, during their blooming season; between October and December [5, 7, 8]. Kraalbos is considered a highly invasive pioneer plant, being the first perennial to regrow after soil disturbances, and the only remaining species after the veld has been heavily overgrazed. Some local farmers claim that during the summer months the plant is poisonous to goats and sheep [4]. According to other farmers, if kraalbos is green, it is palatable and not poisonous, but if it is yellow and dry, it is non-palatable and poisonous [9].

Traditional uses of *G. africana* by the southern African indigenous Khoi-San people involve chewing the plant to relieve toothache or preparing a decoction which is used to make a lotion for the treatment of wounds [10, 11]. It has been shown that frying *G. africana* with other medicinal plants in butter to create an ointment has been used to treat wounds, particularly on the legs of women [10]. Other uses which have been described in ethnobotanical surveys include making a leaf infusion to wash their heads for the purpose of treating pimples and rashes on the affected area. The same infusion was also used in the treatment of dandruff, lice, dry skull, leg pains, and swollen legs. In addition, topical products are prepared for the treatment of venereal diseases as well as skin infections and ailments such as ringworm [6, 12]. There have also been reports of *G. africana* leaves being used for chest pains, and conditions such as asthma and *tuberculosis* [11]. The medicinal potential of botanicals and natural ingredients derived from *G. africana* could be attributed to the plant being a major source of polyphenolic flavonoids and other compounds which are associated with antioxidant activities [13].

The aim of this study is to report on the phytochemical composition of *G. africana* and provide a comprehensive background on the pharmacological importance and relevance of these phytochemicals. The valuable information gained from such a study of the phytochemicals present in *G. africana* will highlight the importance and relevance for treatment of skin diseases described through traditional applications and ethnobotanical surveys. A review on dietary intake of the phytochemicals was used to inform potential health risks after accidental intake of the *G. africana* extract.

## 2. Materials and Methods

### 2.1. Identification of Phytochemicals of Galenia africana

It is important that medicinal plants be analysed to determine their phytochemical constituents, which would be beneficial in understanding the pharmacological importance and health risks. The first preparation of a *G. africana* extracts for antifungal and antibacterial fractionation including phytochemical identification studies was employed by Vries et al. (2005) and Mativandlela et al. (2009) [14, 15]. A study by Ticha et al. (2015) analysed a representative 20% extract (80% ethanol : water) of *G. africana*, obtained from Parceval Pharmaceuticals (Pty) Ltd (South Africa), using gravity liquid column chromatography (GLCC) to afford several fractions, which differ in levels of purity, and using EtOAc : hexane as a gradient eluent (hexane, hexane : EtOAc, and EtOAc) [13]. In this study, six of these fractions (A-F) were received as a gift from Dr. Lawrence Ticha, prepared during his postgraduate studies at the Chemistry Department, University of the Western Cape, and these fractions were subjected to a direct gas chromatography-mass spectrometry (GC-MS) analysis, as described by Al-Asmari et al. (2015) [16]. The GC-MS was performed at the Central Analytical Facilities, University of Stellenbosch, with results illustrated in Table 1. Nuclear magnetic resonance (^1^H and ^13^C NMR) spectra were recorded in either CDCl_3_ or acetone-d6 on a Varian Gemini 2000 spectrometer at 200.05 MHz for ^1^H and 50.3 MHz for ^13^C spectra, respectively. All chemical shifts are expressed in parts per million (ppm) relative to trimethylsilane (TMS) as the internal reference standard. GC-MS analysis was carried out in a GC system (Agilent 7890A series, USA) equipped with split/splitless injector and autosampler attached to an apolar 5-MS (5% phenylpolymethyl siloxane) capillary column (Agilent 19091S-43; 30 *m* × 0.25 mm i.d. and 0.25- *μ*m film thickness) and fitted to mass detector (Agilent 5975C series, USA). The flow rate of the carrier gas, helium (He) was set to be at 1 ml.min−1, split ratio is 1 : 50. The injector temperature was adjusted at 250°C, while the detector temperature was fixed to 280°C. The column temperature was kept at 40°C for 1 min followed by linear programming to raise the temperature from 40° to 120°C (at 4°C min−1 with 2 min hold time), 120°C to 170°C (at 6°C min−1 with 1 min hold time), and 170°C to 200°C (at 10°C min^−1^ with 1 min hold time). The transfer line was heated at 280°C. Two microliters of sample was injected for analysis. Mass spectra were acquired in scan mode (70 eV), in the range of 50 to 550 m/*z* [16].

**Figure 1:**
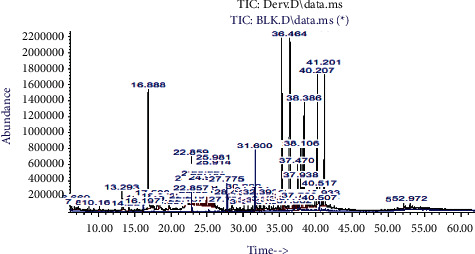
Chromatogram of the *Galenia africana* extract sample.

**Figure 2:**
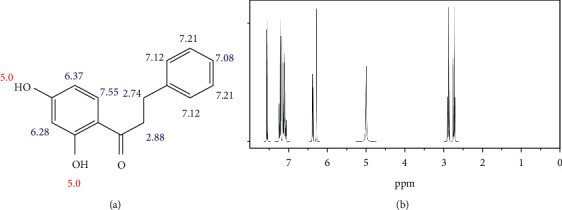
(a) Calculated spectrum and chemical structure of the flavonoid 2',4′-dihydroxydihydrochalcone and (b) 1H NMR spectrum of 2′,4′-dihydroxydihydrochalcone.

Positive identification of compounds was made by matching mass spectra against a reference library database, with results illustrated in the chromatogram (Figure 1) and compound structures in Table 1. Various flavonoids, isolated by gravity chromatographic separation and NMR analysis (Figure 2), and reported by Ticha et al. (2015) [13], are also included in Table 1. These clearly demonstrate that flavonoids were the major secondary plant metabolites present and possible activity was expected to result from these compounds as several previous studies have shown these compounds to have beneficial effects [13]. The relative concentration percentage (%) of any compound was calculated by the individual GC-MS peak area divided by the total peak area of the fraction and multiplying the result by 100.

### 2.2. Dietary Risk Analysis

APC Pharmaceuticals and Chemicals, United Kingdom, was contracted by the University of the Western Cape to conduct a toxicological review and health risk assessment of the components of the *G. africana* extract for agriculture operators and consumers. Since the extract is composed of about 61 compounds, most of which are already present in the human diet, an alternative to conventional toxicity testing procedures was proposed, thereby reducing laboratory animal use and minimising development costs and timeline. An alternative proposed by APC was to utilise peer reviewed literature to assess background dietary exposures for each component. The background dietary intake of the constituents of *G. africana* was analysed using the European Union Pesticide Residue Intake Model (EU PRIMO) consumer risk assessment model which informed a risk assessment for consumers. All compounds approved by the risk assessment were deemed to be nontoxic at the indicated concentration intakes and therefore did not pose a risk to human health, except cinnamic acid, the coumarins, equol, and 2-methoxy-4-vinyl phenol. The dietary intakes for the phytochemicals, as far as could be established from the literature, are summarised and referenced in Table 1 and the discussion.

### 2.3. Literature Search

An initial literature search was conducted with a combination of keywords such as *Galenia africana*, distribution, traditional use, medicinal uses, ethnomedicinal uses, phytochemistry, and chemical composition. After data for the phytochemical composition of *Galenia africana* was established, an extended literature search was performed to determine the natural occurrence and phytochemical and toxicological relevance of the individual constituents. This was done using a combination of the phytochemical name and keywords such as pharmacology, antioxidant, antiproliferative, antifungal, antibacterial, anti-inflammatory, wound healing, and toxicity. These searches were conducted using major databases, including Science Direct, Google Scholar, BioMed Central (BMC), Web of Science, Springer link, Scopus, and PubMed.

## 3. Results and Discussion

Results about the phytochemical composition of *Galenia africana* extract fractions and dietary intake risk in humans are shown in the chromatogram (Figure 1) and Table 1. Figure 2 details the calculated spectrum and 1H NMR spectrum of the flavonoid 2′,4′-dihydroxydihydrochalcone.

## 4. Natural Occurrence and Pharmacological Relevance of Phytochemicals Identified in the *Galenia Africana* Extract

The components contained in the *G. africana* extracts and their relative concentrations are listed in Table 1. Additionally, each of these phytochemical constituents was placed into one of the following groups: aliphatics, aliphatic triterpenoids, fatty acids, flavonoids, tocopherols, and phenolic compounds. The natural occurrence and pharmacological relevance for the compounds as far as could be established from the literature are outlined below.

### 4.1. Aliphatics

Aliphatics are a class of hydrocarbons, where medium and long chain alkanes are components of the surface waxy cuticle of plants and fruits. The function of this waxy layer is to control the rate of moisture loss and to provide a first line of defence against pathogenic organisms [34]. Humans are also exposed to paraffins and waxes from industrial origin, mainly via transfer of these compounds from packaging materials and through the pharmaceutical uses of paraffins. Heptacosane, nonacosane, and pentacosane have all been shown to possess antibacterial activity [35]. Hexacosane extracted from *Sanseveria liberica* was shown to exhibit moderately high antimicrobial activities against *Salmonella typhi, Candida albicans, Streptococcus pyogenes, Staphylococcus aureus, Escherichia coli, Pseudomonas fluorescence, Klebsiella pneumoniae, Proteus vulgaris*, and *Candida krusei* [36]. Due to limited information from the literature, specific pharmacological relevance of the remaining compounds classed under the aliphatic group could not be determined.

### 4.2. Aliphatic Triterpenoids

Farnesol, farnesyl acetone, hexahydrofarnesyl acetone, pristine, and squalene are all classified as aliphatic triterpenoids. These are commonly found in plant matter and fruits which are frequently consumed by humans. Squalene is an aliphatic triterpenoid, present in many foods including amaranth oil and olive oil. In human skin physiology, squalene is used as an antioxidant and moisturizer. It has also been reported to be used for treating skin disorders such as acne, atopic dermatitis, psoriasis, and seborrheic dermatitis [27]. Farnesyl acetone is found in the medicinal herb *Costus pictus*, also in tomato and watermelon [37]. In mushrooms, farnesyl acetone has been detected as a volatile component, accounting for up to 1% of volatiles in *Suillus granulatus* and up to 12% of *Suillus luteus* [29, 30]. Farnesol has been detected in plants and fungi, accounting for up to 16% of total volatiles in *Chroogomphus rulitus* [38]. However, farnesyl compounds are also endogenous in humans where they are involved in the production of cholesterol and protein tagging [38, 39]. A study conducted by Chaudary et al. (2009) demonstrated the chemopreventative effect of farnesol on skin tumorigenesis [40].

### 4.3. Fatty Acids

Fatty acids are common components found in nature, where they are present in animal or vegetable fats, oils, and waxes [41]. They form chains and are classified according to the length and number of carbon atoms present in the chain, such as short, medium, long, or very long [41]. Humans can synthesise the full range of essential dietary fatty acids used for energy besides the linoleic and alpha linolenic acids. Dietary fatty acids are used for energy through the beta-oxidation pathway in addition to assisting in cell wall synthesis and hormone manufacture [42].

Ethyl hexadecanoate (also known as ethyl palmitate) is the ethyl ester of palmitic acid. It is formed in humans in the nonoxidative metabolite pathway of ethanol and has been proposed as a biomarker for ethanol consumption [43]. It has also been listed as an emollient used in cosmetic products [44]. Ethyl tetracosonate is the ethyl ester of tetracosanoic acid which is present in peanut oil accounting for 1.1–2% mass fraction of total fatty acids [25]. Ethyl linoleate and ethyl linolenate are the ethyl esters of linoleic and alpha linolenic acid, respectively. According to PubChem (2021), hexadecanoic acid (palmitic acid) is used as an emollient in cosmetic products [45]. A study by Uddin et al. (2012) demonstrated that tetracosane had significant cytotoxic activity against HT-29 colon cancer cells and some toxicity against gastric cancer and estrogen-dependant breast cancer cells [46]. A recent study by Gao et al. (2019) demonstrated the use of hexanedioic acid (adipic acid) as a component of a hydrogel which shows potential for wound healing applications [47]. Additionally, adipic acid is used in cosmetics as a buffering agent [48].

Linoleic acid ethyl ester (ethyl linoleate) is an unsaturated fatty acid resulting from formal condensation of the carboxyl group of linoleic acid with the hydroxyl group of ethanol. It is used in many cosmetic products for its antibacterial and anti-inflammatory properties [49]. A recent study by Ko and Cho (2018) demonstrated the potential of ethyl linoleate as a noncytotoxic and skin whitening agent in medicine and cosmetic products [50]. Pentadecanoic acid has an uneven number of carbon atoms and is thought to be synthesised primarily by gut microflora in the rumen of cattle. Hence, the major dietary source of this fatty acid is milk, where it accounts for approximately 1% of milk fats [51]. It has been shown that pentadecanoic acid could serve as a signalling inhibitor in breast cancer cells [52]. Tetradecanoic acid (also known as myristic acid) is present in coconut oil and palm kernel fat accounting for approximately 16% mass fraction of total fatty acids [25]. It is also present in milk (11% mass fraction of total fatty acid), meat, and meat products [25]. Tetradecanoic acid is used as a cleansing and emulsifying agent in cosmetic products [53].

### 4.4. Flavonoids

The flavonoids comprise a group of low molecular weight compounds, of which roughly 4000 are known, and are separated into distinct subgroups: anthocyanidins, chalcones, flavanols, flavanones, flavones, isoflavones, and stannols [54]. They are ubiquitous in plants and have several biological functions. For example, they are responsible for pigmentation of many plants and fruits [55]. The flavonoids, *trans*-Cinnamic acid, chalcone, chrysin, and tectochrysin, are associated with a variety of health-promoting effects and are an important component in many nutraceutical, pharmaceutical, medicinal, and cosmetic applications [56–60]. This is due to their anticarcinogenic, anti-inflammatory, antimutagenic, and antioxidative properties [55]. Flavonoids are not classified as essential human nutrients per se, but a high dietary intake of flavonoids is thought to be associated with lowered cancer and cardiovascular disease risk [54]. 2,4-Dihydroxychalcone is a flavonoid abundant in the leaves of *Oxytropis falcata*, a leguminous plant also known as locoweed. This plant has been widely used in Chinese/Tibetan herbal medicine. In addition to its antioxidant activity, it also exhibits antitumor activity [61, 62]. Equol is the major intestinal bacterial metabolite of the isoflavanone diadzin in 25–60% of the population and is influenced by diet [63]. Those consuming a diet high in diadzin (e.g., vegetarian diets and/or those containing soya) were more likely to metabolise the material to equol [63]. Equol has been shown to have antiaging properties and antiandrogen activity [64, 65]. Naringenin is found in many citrus fruits and has been measured in grapefruit juice at concentrations of 100–800 mg/L [23]. There is growing evidence showing the pharmacological effects of naringenin which include anticancer, anti-inflammatory, antimicrobial, antimutagenic, and hepatoprotective properties [66]. Pinocembrin is a major flavanone found in honey and propolis, the resinous substance used by bees to seal honeycombs and used as a health food [67]. Pinocembrin has shown potential for use as an antioxidative, anti-inflammatory, antimicrobial, and antitumor agent [68].

### 4.5. Phenolic Compounds

Results reveal 2-methoxy-4-vinylphenol to be present in many products where production involves the use of yeast and/or cooking. It is also a component of beer, especially wheat beer, formed by the conversion of ferulic acid during the fermenting process [69]. In a study by Kim et al. (2019), it was shown that 2-methoxy-4-vinylphenol possesses anticancer properties and exhibits antiaging and antioxidant activity [70, 71].

### 4.6. Tocopherols

Alpha-tocopherols are part of a group of fat-soluble compounds known as vitamin E. Along with being the most common form of vitamin *E* present in nature, alpha-tocopherols are also the most biologically active [72]. These can be found in foods such as avocados, nuts, and seeds. In a study by Weber et al. (1997), it was shown that a topical application of alpha-tocopherol to mouse skin prior to exposure of UV-irradiation resulted in the preservation of antioxidants [57, 73]. Conversely, without prior application of the tocopherol, the antioxidants present in the skin were destroyed after the irradiation. Alpha-tocopherolquinone is a metabolite of alpha-tocopherol [74]. Vitamin *E* derivatives such as alpha-tocopherolquinone act as an important physiological antioxidant. Alpha-tocopherolquinone has further demonstrated its potential as a biomarker for oxidative stress [75].

Vitamin *E* is an essential nutrient obtained by external sources such as fresh vegetables, vegetable oils, cereals, and nuts. Vitamin *E* has been demonstrated to be of importance in recent dermatological studies due to its antioxidant properties. Experimental evidence suggests that topical application and oral consumption of vitamin *E* have anticarcinogenic, photoprotective, and skin barrier-stabilizing properties [76]. Vitamin *E* acetate has elicited a significant interest for its role in assisting in curing burn injuries, particularly for its antioxidant action which occurs during tissue reperfusion. Reactive oxygen species and free radicals are produced during the phase of reperfusion of ischemic tissues, damaging numerous cell components, including nucleic acids, lipids, and proteins. It has been shown that a bioadhesive film containing vitamin *E* acetate could facilitate skin regeneration and wound healing through the controlled release of the vitamin *E* acetate [77].

## 5. Potential Health Risks of Phytochemicals Identified in the *Galenia africana* Extract

Information from the US Dietary Reference Intake (DRI), Joint FAO/WHO Expert Committee on Food Additives (JECFA), and Beare-Rogers et al. (2009) IUPAC Technical Report was used to establish average daily background dietary intake values (Table 1) [18,25,78]. The background dietary intake of the constituents of *G. africana* was analysed using the European Union Pesticide Residue Intake Model (EU PRIMO) consumer risk assessment model, which informed a risk assessment for consumers [79, 80].

Based on the *G. africana* phytochemical data, it was observed that most compounds were already present in the general human diet and therefore should not pose a risk to human health. Potentially relevant components, besides cinnamic acid, the coumarins, equol, and 2-methoxy-4-vinyl phenol, were all found to be nontoxic following preliminary chronic and acute consumer risk assessments. The dietary intakes and potential health risks for the phytochemicals in Table 1, as far as could be established from the literature, are summarised below.

For aliphatics in general, Tennant (2004) has estimated that the average intake of each of the mixed alkanes is 0.01–0.02 mg/kg/day [81]. Based on this data and information from the European Food Safety Authority, which considered and listed paraffin oils for use as a pesticide active ingredient, it was deemed that no risk assessment would be necessary as the straight chain alkanes were toxicologically nonrelevant.

The aliphatic triterpenoid, squalene, is especially prevalent in olive oils, and as a result human dietary intake varies greatly according to the geographical location where intake is estimated at 30 mg/day in the USA but increases to 200–400 mg/day in Mediterranean countries [26]. No quantitative information was available regarding the dietary intakes of faresyl acetone and hexahydrofarnesyl. However, hexahydrofarnesyl acetone undergoes beta-oxidation and is unlikely to pose greater hazards than fatty acids or aliphatics. Farnesyl acetone intake can be approximated as it is approved as a flavouring agent by JECFA with an estimated daily intake of 9 µg/day.

According to Madigan et al. (1994), the phenolic compound 2-methoxy-4-vinylphenol is present in wheat beer at a concentration of 0.68 mg/L [82]. The average Czech Republic intake of beer is 156 L/year giving rise to an average daily intake of 2-methoxy-4-vinylphenol from beer at 0.29 mg/day. Consuming 2 L of beer in one day, as might occur occasionally, results in consumption of 1.36 mg/day.

According to Kennedy *et al.*, (1999) the dietary intake of fatty acids in the USA is between 41 and 117 g/day with saturated fat accounting for 14–42 g of this amount [55, 83]. Given that fatty acids are a significant part of the normal human diet, they are not toxicologically relevant components of the extract and may be excluded from risk assessments.

The potential link between flavonoid intake and health has led to a number of studies measuring the dietary intake of flavonoids in humans. These have produced differing results ranging from 23 mg/day to 1 g/day [84–86]. As such, the potential dietary intake of the many flavonoids may account for up to 600 mg/day if all flavonoids are considered.

As a tocopherol, the recommended daily intake of vitamin *E* has been set by many countries. According to the US DRI of alpha-tocopherol, intake is set at 15 mg/day with an upper tolerable intake of 1,000 mg/kg [87].

## 6. Conclusions

*Galenia africana* is a plant of traditional medicinal and commercial relevance in South Africa. In this review, the importance and relevance of the phytochemical constituents and potential risks relating to exposure to the *G. africana* extract were concluded in a descriptive manner. Phytochemical screening results indicate the relative chemistry and concentrations of the compounds in the plant extract. Literature also shows that several compounds present in the *G. africana* extract exhibit anti-inflammatory, antioxidant, and antimicrobial activities and wound healing which supports the traditional medicinal use of the plant by indigenous people. Furthermore, it was revealed that none of the potentially relevant compounds in *G. africana* were expected to cause undue risk to human health. This data regarding the potential risks related to the ingestion of *G. africana* extract could be used to infer the potential risks relating to the accidental exposure to these phytochemical compounds when exposed to human skin upon topical application. On this basis, the effect of the *Galenia africana* extract on human skin in quantities similar to those described in this review should not cause undue risk to human health. However, it is important that further studies be performed using this plant to determine pharmacological action and mechanism of action, which has the potential to lead to the development of *G. africana* as a therapeutic agent.

## Figures and Tables

**Table 1:** Phytochemical composition of *Galenia africana* extract fractions and dietary risk intake risk in humans.

CAS number^+^	Chemical structure^*∗*^	Common or IUPAC name	Relative concentration (%)^++^	Chemical class	Dietary intake (mg/day)^+++^
*Flavonoids in 20% extract*					
305-01-1	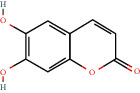	Chrysin or aesculetin	0.54	Flavonoid	0.008 [17]
1776-30-3	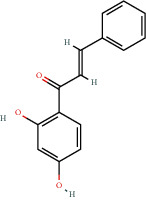	2′,4′-Dihydroxychalcone	14.00	Flavonoid	300 [18]
480-39-7	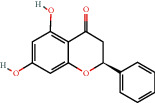	5,7-dihydroxyflavanone/dihydrochrysin/Pinocembrin)	23.10	Flavonoid	0.0043 [17,19]
480-44-4	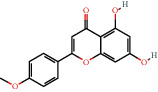	5,7-Dihydroxy-4′-methoxyflavone/Acacetin	0.40	Flavonoid	0.4 [20]
531-95-3	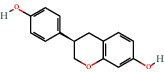	Equol/4′,7-isoflavandiol	3.87	Flavonoid	9.9 [21,22]
6665-86-7	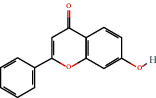	7-Hydroxyflavone	10.20	Flavonoid	No data
520-28-5	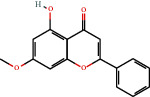	5-Hydroxy-7-methoxyflavone (tectochrysin)	7.07	Flavonoid	0.001 [17]
67604-48-2	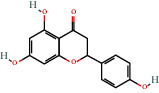	5,7,4′-trihydroxyflavanone/naringenin	0.44	Flavonoid	45 [23]

*Fraction a obtained from 20% extract*					
88-99-3	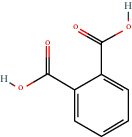	Benzene 1,2-dicarboxylic acid (mono 2-ethylhexyl ester)	0.65	Other	30 [24]
628-97-7	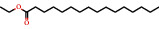	Ethyl hexadecanoate	1.88	Fatty acid	5000 [25]
24634-95-5	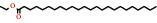	Ethyl tetracosanoate (C24 ester)	0.58	Fatty acid	100 [25]
593-49-7	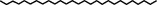	Heptacosane	5.98	Aliphatics	56.4 [18]
7796-19-2	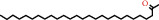	2-Heptacosanone	0.55	Aliphatics	56.4 [18]
630-01-3	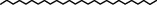	Hexacosane	1.12	Aliphatics	56.4 [18]
544-76-3	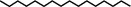	Hexadecane	0.77	Aliphatics	56.4 [18]
103-23-1	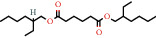	Hexanedioic acid, bis(2-ethylhexyl) ester	0.96	Fatty acid	5 [25]
544-35-4	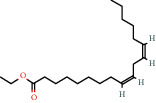	Linoleic acid ethyl ester	1.01	Fatty acid	17000 [18]
630-03-5	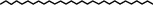	Nonacosane	10.36	Aliphatics	56.4 [18]
593-45-3	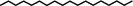	Octadecane	0.80	Aliphatics	56.4 [18]
1191-41-9	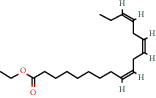	9,12,15-Octadecatrienoic acid, ethyl ester (ZZZ)-	0.85	Fatty acid	1600 [18]
629-99-2	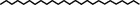	Pentacosane	0.90	Aliphatics	56.4 [18]
1117-52-8	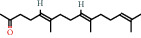	5,9,13-Pentadecatrien-2-one- (6,10,14)-trimethyl (E,E)	1.28	Aliphatic triterpenoid	0.540 [24]
111-02-4	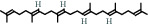	Squalene	0.62	Aliphatic Triterpenoid	400 [26,27]
646-31-1	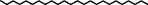	Tetracosane	3.11	Aliphatics	56.4 [18]
638-68-6	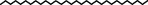	Triacontane	0.66	Aliphatics	56.4 [18]
502-69-2	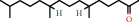	6,10,14-Trimethyl-2-pentadecanone	1.72	Aliphatics	0.540 [18]

*Fraction B obtained from 20% extract*					
117-81-7	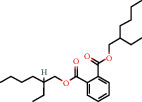	1,2-Benzene dicarboxylic acid mono 2-ethylhexyl ester	2.19		30 [24]
120-51-4	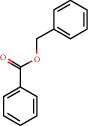	Benzylbenzoate	1.52	Other	300 [18]
103-23-1	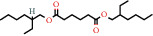	Bis (2-ethylhexyl) hexanedioic acid	1.62		300 [18]
628-97-7	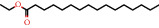	Ethyl hexadecanoate	1.35	Fatty acid	5000 [25]
1191-41-9	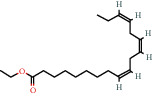	Ethyl Z,Z,Z- 9,12,15-octadecatrienoate	1.03	Fatty acid	1600 [18]
4602-84-0	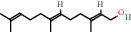	Farnesol	3.56	Aliphatic triterpenoid	0.009 [28–30]
103-30-0	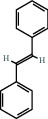	(E)-stilbene	1.45	Flavonoid	200 [31]
646-31-1	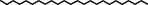	Tetracosane	0.79	Aliphatics	56.4 [18]
		*α*-Tocopherolquinone	1.15	Tocopherol	15 [18]
502-69-2	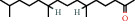	6,10,14-Trimethyl-2-pentadecanone	5.29	Aliphatics	0.540 [24]
10191-41-0	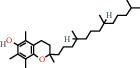	Vitamin E	1.33	Tocopherol	15 [18]
58-95-7	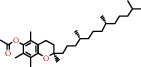	Vitamin E-acetate	1.71	Tocopherol	15 [18]

*Fraction C obtained from 20% extract*					
140-10-3	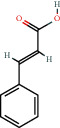	*trans*-Cinnamic acid	0.32	Flavonoid	2.7 [24]
6538-02--9	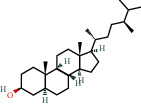	Ergostanol	0.42	Flavonoid	Not absorbed
57-10-3	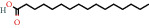	Hexadecanoic acid	0.49	Fatty acid	5000 [18]
1002-84-2	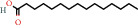	Pentadecanoic acid	1.25	Fatty acid	100 [25]
122-57-6	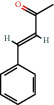	4-Phenylbut-3-en-2-one	0.24	Carbonyl	No data
544-63-8	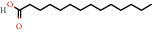	Tetradecanoic acid	0.19	Fatty acid	1300 [25]
502-69-2	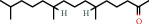	6,10,14-Trimethyl-2-pentadecanone	1.25	Aliphatics	0.540 [24,32]
10191-41-0	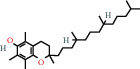	Vitamin E	0.20	Tocopherol	15 [18]

*Fraction D obtained from 20% extract*					
92-48-8	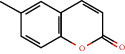	2H-1-benzopyran-2-one	0.39	Flavonoid	No data
305-01-1	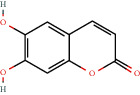	Esculetin	0.46	Flavonoid	6.0 [18]

Fraction *E* obtained from 20% extract					
140-10-3	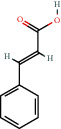	*trans*-Cinnamic acid	0.47	Flavonoid	2.7 [24]
305-01-1	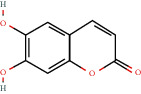	Esculetin	1.05	Flavonoid	6.0 [18]
544-76-3	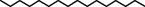	Hexadecane	0.32	Aliphatics	56.4 [18]
629-59-4	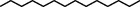	Tetradecane	0.22	Aliphatics	56.4 [18]
126-33-0	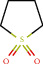	Tetrahydrothiophene 1,1-dioxide	1.03	Other	No data

Fraction F obtained from 20% extract					
140-10-3	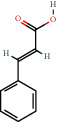	*trans*-Cinnamic acid	2.05	Flavonoid	2.7 [24]
1776-30-3	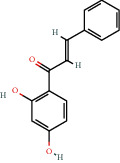	2,3-Dimethoxy-2′,4′-dihydroxy chalcone	2.86	Flavonoid	300 [18]
23470-00-0	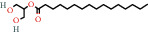	Ethylhexadecanoic acid-2-hydroxy ester	3.46		5000 [19,25]
544-76-3	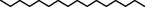	Hexadecane	0.83	Aliphatics	56.4 [18]
7786-61-0	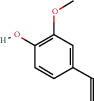	2-Methoxy-4-vinyl phenol	0.94	Phenolic	0.29 [33]
593-45-3	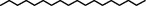	Octadecane	1.19	Aliphatics	56.4 [18]
784-62-3	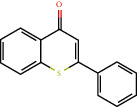	2-Phenyl-4-H-1-benzothiopyran-4-one	2.73	Other	No data
629-59-4	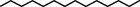	Tetradecane	0.81	Aliphatics	56.4 [18]
126-33-0	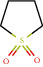	Tetrahydrothiophene 1,1-dioxide	8.92	Other	No data

^a^Direct GC-MS analysis of chromatography fractions of differing purity after CC fractionation of 20% *G. africana* extract. ^b^ Mass spectrum of compounds match with 360000 memory banks. ^+^The CAS number is a unique numerical identifier assigned by the Chemical Abstracts Service (CAS) to chemical substances described in the open literature to create a link to information about a specific chemical substance. ^++^The relative concentration percentage (%) of any compound was calculated by the individual GC-MS peak area divided by the total peak area of the fraction and multiplying the result by 100. ^+++^The dietary intake data for the phytochemicals was obtained from data found in peer reviewed articles and reference databases. ^*∗*^Chemical structures were obtained by using the CAS number to search for the chemicals on the PubChem database.
